# Good perceived sleep quality protects against the raised risk of respiratory infection during sleep restriction in young adults

**DOI:** 10.1093/sleep/zsac222

**Published:** 2022-09-16

**Authors:** Neil P Walsh, Daniel S Kashi, Jason P Edwards, Claudia Richmond, Samuel J Oliver, Ross Roberts, Rachel M Izard, Sarah Jackson, Julie P Greeves

**Affiliations:** Faculty of Science, Liverpool John Moores University, Liverpool, UK; Faculty of Science, Liverpool John Moores University, Liverpool, UK; Faculty of Science, Liverpool John Moores University, Liverpool, UK; Faculty of Science, Liverpool John Moores University, Liverpool, UK; College of Human Sciences, Bangor University, Bangor, UK; College of Human Sciences, Bangor University, Bangor, UK; Defence Science and Technology, Porton Down, UK; Army Health and Performance Research, Army HQ, Andover, UK; Army Health and Performance Research, Army HQ, Andover, UK; Norwich Medical School, University of East Anglia, Norwich, UK

**Keywords:** sleep, sleep restriction, sleep quality, sleep duration, sleep debt, infection

## Abstract

**Study Objectives:**

Prospectively examine the association between sleep restriction, perceived sleep quality (PSQ) and upper respiratory tract infection (URTI).

**Methods:**

In 1318 military recruits (68% males) self-reported sleep was assessed at the beginning and end of a 12-week training course. Sleep restriction was defined as an individualized reduction in sleep duration of ≥2 hours/night compared with civilian life. URTIs were retrieved from medical records.

**Results:**

On commencing training, approximately half of recruits were sleep restricted (52%; 2.1 ± 1.6 h); despite the sleep debt, 58% of recruits with sleep restriction reported good PSQ. Regression adjusted for covariates showed that recruits commencing training with sleep restriction were more likely to suffer URTI during the course (OR = 2.93, 95% CI 1.29–6.69, *p* = .011). Moderation analysis showed this finding was driven by poor PSQ (*B* = −1.12, SE 0.50, *p* = .023), as no significant association between sleep restriction and URTI was observed in recruits reporting good PSQ, despite a similar magnitude of sleep restriction during training. Associations remained in the population completing training, accounting for loss to follow-up. Recruits reporting poor PSQ when healthy at the start and end of training were more susceptible to URTI (OR = 3.16, 95% CI 1.31–7.61, *p* = .010, *vs* good PSQ).

**Conclusion:**

Good perceived sleep quality was associated with protection against the raised risk of respiratory infection during sleep restriction. Studies should determine whether improvements in sleep quality arising from behavioral sleep interventions translate to reduced respiratory infection during sleep restriction.

## Graphical Abstract

**Graphical Abstract:**
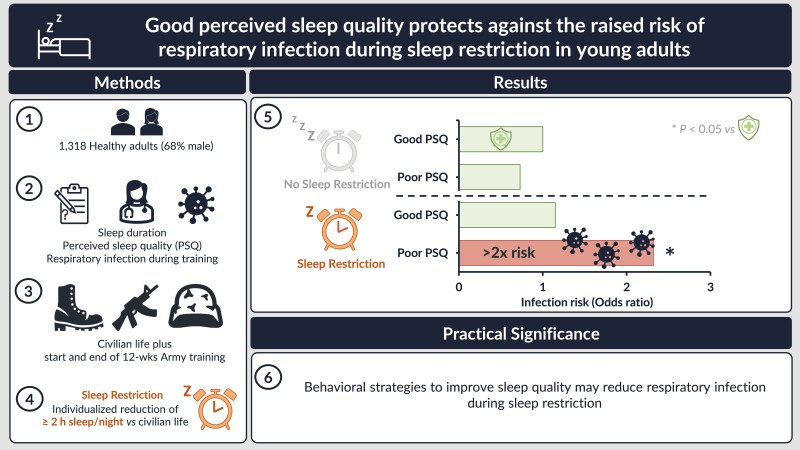

**Statement of Significance**

Restricting habitual sleep to accommodate the demands of modern life is now commonplace (e.g., rising early for work). Whether sleep restriction, an individualized reduction in sleep ≥2 hours/night, increases respiratory infection and good perceived sleep quality provides any protection remains unknown. In civilians embarking on a military career, we show that sleep restriction (*vs* civilian life) increased the risk of physician-diagnosed respiratory infection but only in those reporting poor not good perceived sleep quality. Good perceived sleep quality was associated with protection against respiratory infection during sleep restriction. Future studies should explore the potential for prophylactic effects of nonpharmacological interventions to improve sleep quality and reduce respiratory infection during sleep restriction.

## Introduction

To meet the competing demands of work, family and leisure it is now commonplace for adults to restrict their sleep, resulting in a high proportion of habitual short sleepers in modern society (e.g. those sleeping <6 hours/night) [1]. Evidence suggests that short sleep is associated with increased morbidity and mortality [2, 3]. For example, habitual short sleep is associated with a raised risk of respiratory infection [4–6] and with diseases associated with inflammation [7], including cardiovascular disease [8] and diabetes [9]. Having said this, meeting the recommended 7–9 hours’ sleep each night [10] is unlikely necessary for everyone to achieve optimal health, inter-individual differences in sleep needs are likely an important consideration [11, 12]. A striking demonstration of this can be found in the results of one large epidemiological investigation, the principal finding of which was that short sleep was associated with pneumonia risk; however, pneumonia risk was only increased in short sleepers who perceived they had inadequate sleep, not in those who perceived their sleep to be adequate [6].

Sleep restriction influences immunity by activating the hypothalamic–pituitary–adrenal (HPA) axis and the sympathetic nervous-system [13]. Laboratory studies of sleep restriction, performed in those who routinely achieve sleep recommendations (7–9 h sleep/night) [10], in which sleep was restricted to 4 hours/night, demonstrate circadian misalignment e.g. disrupted HPA axis regulation of the diurnal cortisol rhythm [14, 15], increased inflammation, e.g. raised circulating interleukin-6 and C-reactive protein [15, 16] and an impaired immune response to vaccination [17]. However, it remains unknown whether these laboratory findings on sleep restriction and immunity translate to increased upper respiratory tract infection (URTI), and the impact that accounting for individual sleep habits among a wider spread of sleepers also warrants enquiry, particularly as ~40% of the US adult population report sleeping <7 or >9 hours/night [18].

Another knowledge gap is whether good perceived sleep quality (PSQ) affords any protection against the purported negative effects of sleep restriction on URTI incidence. Subjective assessment of sleep quality, e.g. PSQ using a four point Likert scale, scored from one very poor to four very good [19], relates to objectively assessed sleep parameters, including sleep onset latency and sleep continuity [20, 21]. Although PSQ is often overlooked as a measure in sleep studies in favor of sleep duration [11], the available evidence supports the notion of health benefits in those reporting good rather than poor PSQ e.g. to cardiovascular and immune health [22, 23]. Whilst the mechanism(s) for a purported beneficial effect of good PSQ on immune health requires elucidation, good PSQ has been associated with more restorative slow wave sleep (SWS) [21], considered the most relevant sleep stage in mediating the effect of sleep on the immune system [12]. Accordingly, SWS activity the night after hepatitis A vaccination was predictive of the antibody response; and reductions in circulating cortisol and increases in growth hormone and prolactin during SWS correlated strongly with the expression of antigen-specific CD4 T cells measured up to one year after vaccination [24]. Analogous are study findings showing that poor PSQ is associated with disrupted circadian cortisol rhythm [25, 26] with likely consequences including dysregulated inflammation and immunity and associated poor health outcomes [27, 28]. From a practical standpoint, should good PSQ provide protection against URTI during sleep restriction, it is encouraging to note that nonpharmacological interventions can improve PSQ [29] and promote SWS [30].

With this information in mind, in a cohort of 1318 healthy young males and females entering basic military training, we prospectively examined the association between sleep restriction (an individualized reduction in sleep duration of ≥2 hours/night compared with civilian life [31]), perceived sleep quality and URTI susceptibility. We hypothesized that sleep restriction would be associated with increased URTI susceptibility, and that good PSQ would afford some protection against URTI in those with sleep restriction.

## Methods

### Setting and participants

The data presented herein were collected as part of a prospective, observational program of research investigating immune health and physical performance in British Army recruits undergoing phase one training; the findings from which have been presented elsewhere [32–35]. Here, we present previously unpublished findings showing the protective effect of good PSQ on URTI susceptibility during sleep restriction.

**Figure 1.**
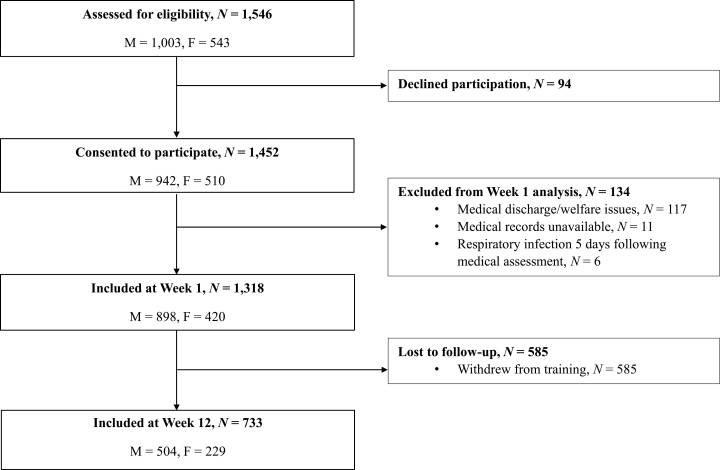
Flowchart outlining recruits assessed for eligibility, consented and available at the start and end of the training course.

A total of *N* = 1546 civilian males and females, ≥18 years of age, entering British Army Phase One Training were assessed for eligibility between January 2014 and November 2015; males were located at the Infantry Training Centre Catterick, UK, and females were located at the Army Training Centre, Pirbright, UK (Figure 1 and Table 1). British Army Phase One Training follows a 12-week syllabus of fundamental military skills including physical training, weapon handling, map reading and fieldcraft. Phase One Training takes place under controlled living and working conditions: recruits reside on the military base in shared living accommodation (up to 12 per room), they all perform largely the same daily activities in groups and eat their meals at a military catering facility, all whilst under the close supervision of their superiors. With the exception of discharge for medical or welfare reasons in Week-1, recruits remain enrolled on the training course, residing on the military base, for at least the first six weeks (Figure 1). After this time weekend leave is permitted and recruits are afforded the opportunity to voluntarily discharge from training. During the busy daily schedule there is scant opportunity for napping and during the evenings there is little opportunity for down time; recruits are required to prioritize this time to complete tasks such as cleaning, ironing, and paperwork over relaxing and using mobile phones. Notwithstanding the rigorous control over day-to-day living and working conditions, including the requirement to arise relatively early in the morning (0500–0630 h), recruits are afforded considerable flexibility over the time they typically retire to bed at night (2100–0100 h). Sleep during civilian life and military training is characterized in Table 2.

**Table 1.** Descriptive information in the population that commenced training.

	Total sample	No sleep restriction			Sleep restriction		
		All	Good PSQ	Poor PSQ	All	Good PSQ	Poor PSQ
	*N* = 1318	*N* = 628	*N* = 420	*N* = 208	*N* = 690	*N* = 400	*N* = 290
*Demographic and lifestyle*							
Age	22 ± 3	22 ± 3	22 ± 3	22 ± 3	21 ± 3^bb^	21 ± 3^aa^	22 ± 3^bcc^
Sex, Male [*N*(%)]	898 (68)	500 (80)	342 (81)	158 (76)	398 (58)^bb^	262 (66)^aa^	136 (47)^aabbcc^
Sex, Female [*N* (%)]	420 (32)	128 (20)	78 (19)	50 (24)	292 (42)^bb^	138 (35)^aa^	154 (53)^aabbcc^
Ethnicity, Caucasian [*N* (%)]	1253 (95)	594 (95)	398 (95)	196 (94)	659 (96)	380 (95)	279 (96)
BMI (kg/m^2^)	23.9 ± 2.7	24.1 ± 2.7	24.1 ± 2.8	24.2 ± 2.7	23.7 ± 2.6^bb^	23.8 ± 2.8	23.6 ± 2.4
Smoker [*N* (%)]	777 (59)	397 (63)	279 (66)	118 (57)^a^	380 (55)^bb^	235 (59)^a^	145 (50)^aac^
*Season of enrolment*							
Winter [*N* (%)]	280 (21)	144 (23)	77 (18)	67 (32)	136 (20)	82 (21)	54 (19)
Spring [*N* (%)]	214 (16)	87 (14)	55 (13)	32 (15)	127 (18)	65 (16)	62 (21)
Summer [*N* (%)]	416 (32)	214 (34)	152 (36)	62 (30)	202 (29)	123 (31)	79 (27)
Fall [*N* (%)]	408 (31)	183 (29)	136 (33)	47 (23)	225 (33)	130(32)	95 (33)
*Total mood disturbance*	5 ± 9	3 ± 8	2 ± 8	6 ± 9^aa^	6 ± 9^bb^	4 ± 9^aa^	8 ± 10^aacc^
*Completed training* [*N (%)*]	733 (56)	365 (58)	248 (59)	117 (56)	368 (53)	218 (55)	150 (52)

Values presented as mean ± SD unless otherwise stated. PSQ = perceived sleep quality; BMI = body mass index. Sleep restriction is defined as an individualized reduction in sleep duration of ≥ 2 hours from civilian life. ^a^ = significantly different to no sleep restriction Good PSQ; ^b^ = significantly different to equivalent no sleep restriction group. ^c^ = significantly different to sleep restriction Good PSQ. Single letter denotes *p* < .05 (e.g. ^a^); double letter denotes *p* < .01 (e.g. ^aa^).

**Table 2.** Sleep characteristics in the population that commenced and completed training.

	Total sample	No sleep restriction			Sleep restriction		
		All	Good PSQ	Poor PSQ	All	Good PSQ	Poor PSQ
*Population that commenced training*	*N* = 1318	*N* = 628	*N* = 420	*N* = 208	*N* = 690	*N* = 400	*N* = 290
Civilian life Duration (h)	8.5 ± 1.5	7.3 ± 1.0	7.3 ± 1.0	7.4 ± 1.1	9.5 ± 1.1^bb^	9.6 ± 1.1^aa^	9.4 ± 1.2^aabb^
Bed/waketime	23:24–07:56	23:22–06:36	23:21–06:36	23:26–06:35	23:26–09:04	23:27–09:11	23:25–08:53
Start of training Duration (h)	6.4 ± 0.8	6.6 ± 0.7	6.7 ± 0.6	6.4 ± 0.7^aa^	6.2 ± 0.8^bb^	6.4 ± 0.6^aa^	6.0 ± 0.9^aabbcc^
Bed/waketime	22:56–05:23	22:46–05:26	22:43–05:27	22:55–05:24	23:05–05:21	22:54–05:21	23:15–05:15
Sleep restriction (h)	2.1 ± 1.6	0.7 ± 1.0	0.6 ± 1.0	1.0 ± 0.9^aa^	3.3 ± 1.1^bb^	3.2 ± 1.0^aa^	3.4 ± 1.2^aabb^
*Population that completed training*	*N* = 733	*N* = 365	*N* = 248	*N* = 117	*N* = 368	*N* = 218	*N* = 150
Civilian life Duration (h)	8.4 ± 1.6	7.3 ± 1.0	7.3 ± 0.9	7.2 ± 1.2	9.6 ± 1.1^bb^	9.7 ± 1.0^aa^	9.4 ± 1.2^aabb^
Bed/waketime	23:21–07:50	23:21–06:34	23:16–06:29	23:32–06:45	23:21–08:56	23:27–09:10	23:11–08:36
Start of training Duration (h)	6.4 ± 0.8	6.6 ± 0.7	6.7 ± 0.6	6.4 ± 0.7^a^	6.2 ± 0.9^bb^	6.4 ± 0.7^aa^	6.0 ± 1.0^aabbcc^
Bed/waketime	22:58–05:24	22:47–05:25	22:45–05:25	22:53–05:25	23:07–05:22	22:55–05:20	23:25–05:25
Sleep restriction (h)	2.0 ± 1.7	0.7 ± 0.9	0.6 ± 0.9	0.8 ± 1.0	3.4 ± 1.1^bb^	3.3 ± 1.0^aa^	3.4 ± 1.2^aabb^
During training Duration (h)	7.0 ± 0.8	7.0 ± 0.7	7.0 ± 0.7	7.1 ± 0.9	7.0 ± 0.9	7.0 ± 0.8	7.0 ± 1.0
Bed/waketime	22:31–05:33	22:31–05:33	22:33–05:32	22:28–05:35	22:31–05:34	22:32–05:34	22:30–05:33
Sleep restriction (h)	1.4 ± 1.3	0.3 ± 1.1	0.3 ± 1.1	0.1 ± 1.3	2.6 ± 1.3^bb^	2.7 ± 1.3^aa^	2.4 ± 1.3^aabb^
End of training Duration (h)	7.0 ± 1.2	6.9 ± 1.1	6.9 ± 1.2	7.0 ± 1.1	7.1 ± 1.3^b^	7.0 ± 1.2	7.3 ± 1.3^a^
Bed/waketime	22:51–05:58	22:53–05:51	22:52–05:47	22:57–06:01	22:54–06:04	22:54–05:55	22:55–06:16
Sleep restriction (h)	1.4 ± 1.9	0.4 ± 1.4	0.4 ± 1.3	0.2 ± 1.4	2.5 ± 1.7^bb^	2.7 ± 1.6^aa^	2.1 ± 1.8^aabbcc^

Values presented as mean ± SD unless otherwise stated. PSQ = perceived sleep quality. Sleep restriction is defined as an individualized reduction in sleep duration of ≥ 2 hours from civilian life. ^a^ = significantly different to no sleep restriction Good PSQ; ^b^ = significantly different to equivalent no sleep restriction group. ^c^ = significantly different to sleep restriction Good PSQ. Single letter denotes *p* < .05 (e.g. ^a^); double letter denotes *p* < .01 (e.g. ^aa^).

### Procedures and measures

A total of *N* = 1452 healthy males and females provided written informed consent to participate in the study before completing a medical assessment with a physician either the day after arrival at the military base or the following day. Medical reasons for exclusion from training included sleep and psychiatric disorders, chronic lung diseases and symptoms or treatment for asthma in the past year. Demographic and lifestyle data were collected at the medical at the start of training, and sleep assessments were made at the start and end of training; participant flow is summarized in Figure 1. Ethical approval was obtained from the UK, Ministry of Defence Research Ethics Committee (reference: 165/Gen/10), with all protocols conducted in accordance with the 2013 Declaration of Helsinki.

### Sleep and mood

At the medical assessment, recruits participating in the study were asked to report the time they went to sleep and woke up on a normal night during civilian life and the night prior to the medical assessment. Recruits also provided a PSQ rating for the night before the medical assessment using a four point Likert scale, where 1 = very poor, 2 = poor, 3 = good and 4 = very good, as previously described [19]. To account for an association between disturbed mood and URTI [36], mood in the past week was assessed at the start of training using the Brunel Mood Scale, which measures five negative mood states (anger, tension, confusion, depression, and fatigue) and one positive mood state (vigor), as previously described [37]. In Week-12, recruits were asked to report the time they went to sleep and woke up on a normal night during training and the time they went to sleep and woke up the previous night with a corresponding PSQ rating.

### Identification of URTI cases

Recruits presenting with URTI symptoms during training reported to the medical facility for a physician consultation. At the end of the training course, participant medical records were accessed by a single independent physician to obtain diagnosed URTIs and associated lost training days due to URTI. The physician’s assessment at the Week-1 medical that the recruit was in good health, ready to commence training, mitigates the risk of reverse causation (i.e. ongoing respiratory illness influencing the Week-1 sleep assessment). Additionally, at the statistical analysis stage, we excluded physician-diagnosed URTIs that occurred in the five days following the Week-1 sleep assessment; a step taken because subjective sleep is typically affected in the first few days of a URTI [38]. There were no cases of physician diagnosed URTI in the five days before the Week-12 sleep assessment.

### Statistical analysis

**Figure 2.**
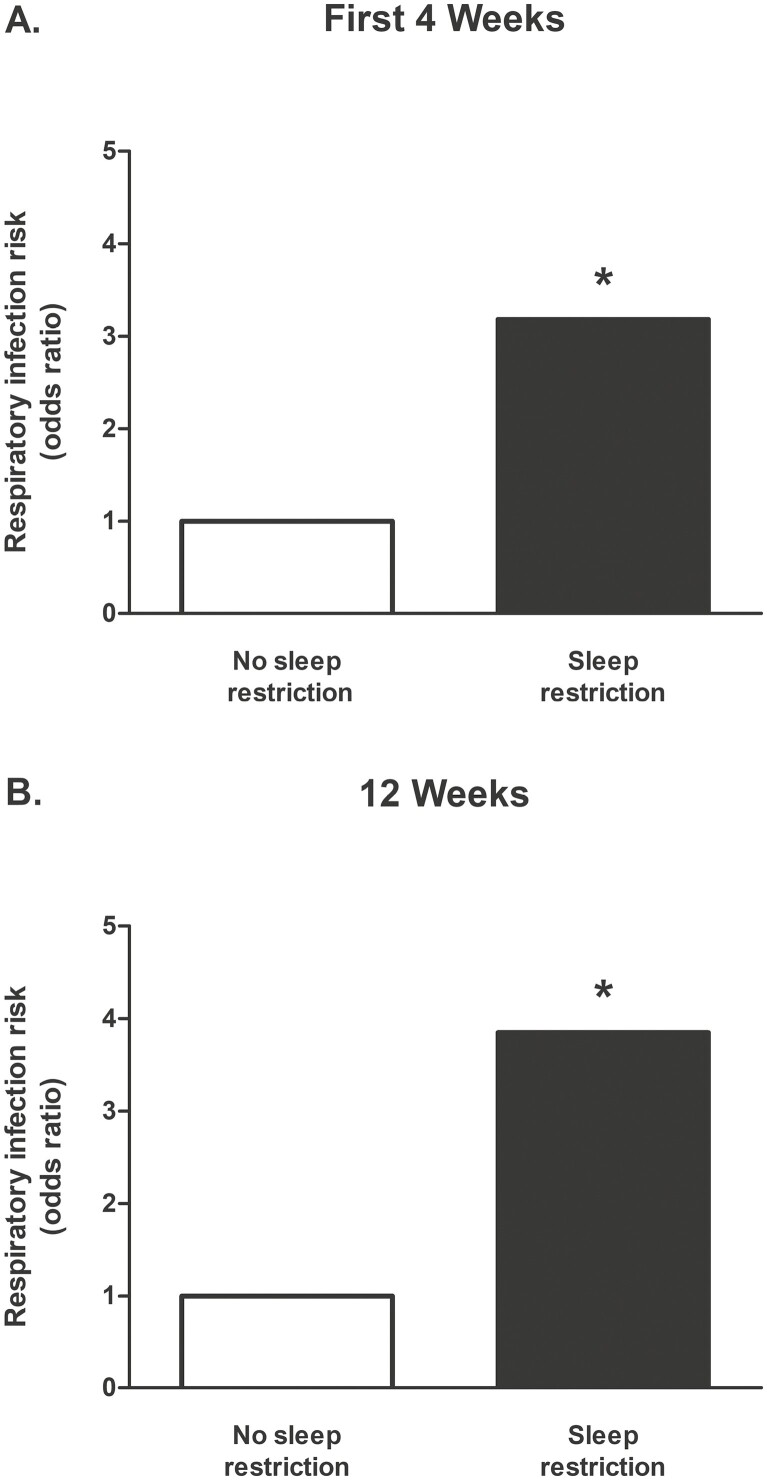
The influence of sleep restriction on URTI susceptibility during the first four weeks of military training (A), and across the 12-week training course in recruits who completed training (B). Sleep restriction is defined as an individualized reduction in sleep duration of ≥ 2 hours from civilian life. Odds ratios presented are from unadjusted analyses. No sleep restriction is considered as reference. URTI = upper respiratory tract infection. * *p* < .05 *vs* no sleep restriction.

All analyses were conducted using SPSS 28.0 (IBM, Armonk, NY, USA) with statistical significance set at *p* < .05. Sleep restriction was defined as an individualized reduction in sleep duration of ≥2 hours/night compared with civilian life Figure 2A–B; studies have chosen two hours as a modest threshold for sleep restriction [31, 39]. As a large proportion of participants reported PSQ ratings of 2 or 3 (81%), a dichotomous grouping was created whereby scores of 1 and 2 were combined to represent “poor” PSQ and scores of 3 and 4 were combined to represent “good” PSQ. Participant demographic, lifestyle and sleep data are presented as mean ± SD for continuous variables or absolute numbers and percentages for categorical variables; comparisons were made using independent t-tests, one-way analysis of variance and Chi-square, where appropriate (Tables 1–3).

**Table 3.** Descriptive information in the populations that completed training and were lost to follow-up.

	Completed training	Lost to follow-up
	*N* = 733	*N* = 585
*Demographic and lifestyle*		
Age	22 ± 3	21 ± 3
Sex, Male [*N* (%)]	504 (69)	394 (67)
Sex, Female [*N* (%)]	229 (31)	191 (33)
Ethnicity, Caucasian [*N* (%)]	699 (95)	554 (95)
BMI (kg/m^2^)	24.0 ± 2.7	23.9 ± 2.7
Smoker [*N* (%)]	433 (59)	344 (59)
*Season of enrolment*		
Winter [*N* (%)]	127 (17)	153 (26)^†^
Spring [*N* (%)]	137 (19)	77 (13)^†^
Summer [*N* (%)]	189 (26)	227 (39)^†^
Fall [*N* (%)]	280 (38)	128 (22)^†^
*Total mood disturbance*	4 ± 8	5 ± 10^†^
*Sleep measures*		
Sleep duration during civilian life (h)	8.4 ± 1.6	8.5 ± 1.4
Bed/waketime	23:21–07:50	23:29–08:04
Sleep duration at the start of training (h)	6.4 ± 0.8	6.4 ± 0.7
Bed/waketime	22:58–05:24	22:54–05:23
Sleep restriction at the start of training (h)	2.0 ± 1.7	2.1 ± 1.5
Sleep quality, Good [*N*(%)]	466 (64)	354 (61)
Sleep quality, Poor [*N*(%)]	267 (36)	231 (39)

Values presented as mean ± SD unless otherwise stated. BMI = body mass index. Sleep restriction is defined as an individualized reduction in sleep duration from civilian life. ^†^*p* < .05 *vs* the population that completed training.

A small proportion of data were missing, assumed missing at random, with 16% (*N* = 211) of recruits missing one or more data points e.g. due to administrative issues with questionnaires. To avoid a potential bias due to excluding incomplete cases, and to maximize statistical power, multiple imputation was carried out using predictive mean matching (50 iterations and 40 imputed datasets), in line with recommendations [40, 41]. All variables were included in multiple imputation, including the outcome variable URTI [40]. The small number of recruits missing medical records (*N* = 11 with no URTI data, Figure 1) were excluded from subsequent statistical analyses, as recommended [42]. Similar demographic and sleep characteristics are shown for complete cases (*N* = 1107) and the dataset following multiple imputation (*N* = 1318; Supplementary Table S1).

**Figure 3.**
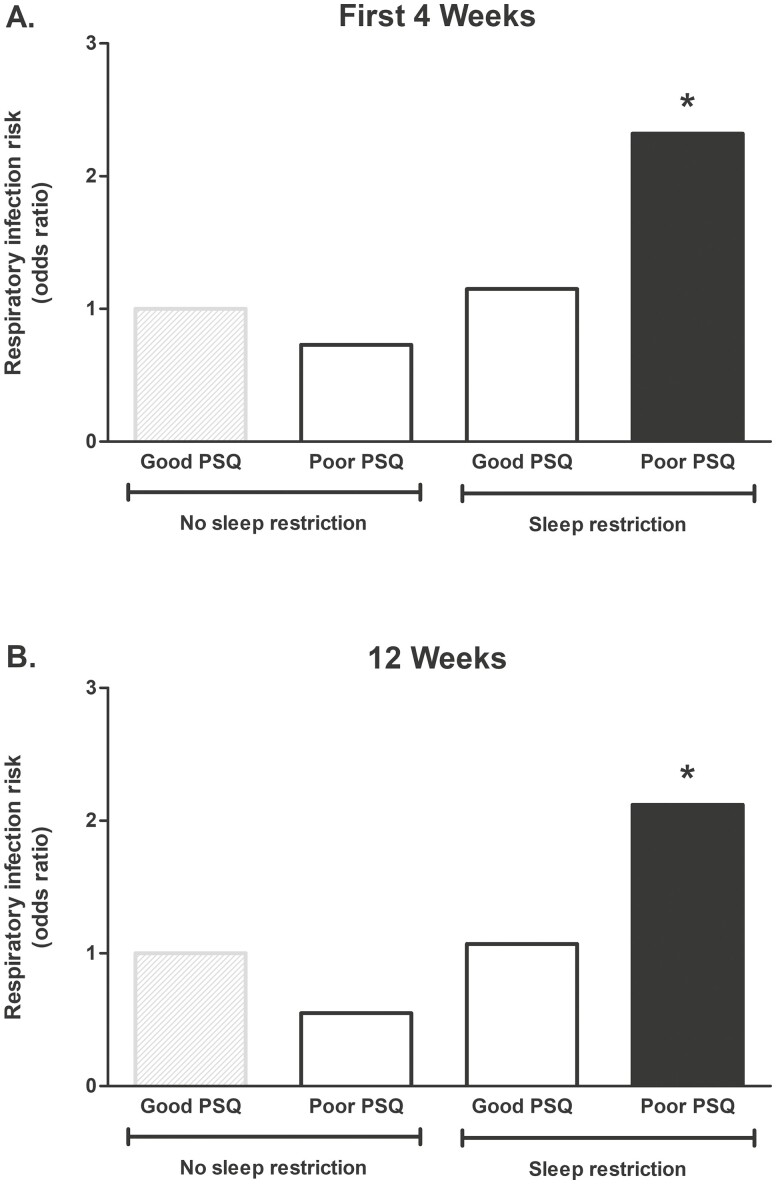
The combined influence of sleep restriction and PSQ on URTI susceptibility during the first four weeks of military training (A), and across the 12-week training course in recruits who completed training (B). Sleep restriction is defined as an individualized reduction in sleep duration of ≥ 2 hours from civilian life. Odds ratios presented are from unadjusted analyses. No sleep restriction and good PSQ considered as reference (grey shaded bar). URTI = upper respiratory tract infection; PSQ = perceived sleep quality. ^*^p < .05 *vs* reference group.

Hierarchical logistic regression was used to examine the moderating effect of PSQ on the association between sleep restriction and URTI susceptibility. After checking assumptions, step 1 examined whether URTI susceptibility was predicted from the independent main effects of sleep restriction Figure 2A–B and PSQ, followed by step 2 examining the interaction effect of sleep restriction and PSQ. Logistic regression was also used to examine the combined influence of sleep restriction and PSQ on URTI susceptibility; whereby, recruits classified as non-sleep restricted who reported good PSQ were considered the reference group (Figure 3A–B and Supplementary Table S3). Separate analyses were used to predict URTI occurring during the first four weeks of training and across the full 12-Week training course. The first four weeks of training was chosen as an additional period of interest because almost half of all URTIs occurred during this period after recruits, traveling from various locations in the United Kingdom, Republic of Ireland and Commonwealth Nations, moved into shared living accommodation (i.e. at a time of heightened pathogen exposure); in addition, recruits were still enrolled in training and remained on the military base during the first four weeks. To account for a selection bias due to loss to follow up, logistic regression was also used to predict URTI cases occurring across the full 12-Week training course in the *N* = 733 who completed training. This population also provided the opportunity to predict URTI susceptibility using logistic regression in a comparative analysis of groups reporting enduring sleep measures e.g. those reporting poor PSQ at both the start and end of training *vs* good PSQ at the start and end of training. For each logistic regression analysis, after checking assumptions, model 1 investigated the unadjusted association with URTI. Adjustment for covariates (likely URTI risk factors) was made as follows: model 2 was additionally adjusted for sex [43] and body mass index (BMI) [44]; model 3 included model 2, plus adjustment for smoking [45]; model 4 included model 3, plus adjustment for season [46]; model 5 included model 4, plus adjustment for total mood disturbance [36]; model 6 included model 5, plus adjustment for long civilian sleep (> 10 h per night) [10]; model 7 included model 5, plus adjustment for short civilian sleep (< 6 h per night) [10]; and model 8 included all previous covariates (fully adjusted model).

## Results

### Sample characteristics, sleep and URTI incidence

Demographic and lifestyle data for the *N* = 1318 recruits who commenced training and the *N* = 733 who completed training are presented in Table 1 and Supplementary Table S2, and sleep characteristics are presented in Table 2. During civilian life, average sleep duration was 8.5 ± 1.5 h (Table 2), in line with recommendations [10], and the proportion of short sleepers (3%, <6 h) and long sleepers (11%, >10 h) was comparable to a representative sample [47]. Accounting for selection bias due to loss to follow-up, demographic, lifestyle and sleep measures were comparable in those who completed the course and those lost to follow-up (Table 3). Moreover, recruits with sleep restriction were no less likely to complete training and the proportion of recruits completing training did not differ across the sleep restriction and PSQ classifications (Table 1).

At the start of training average sleep duration was 6.4 ± 0.8 h, when approximately half of all recruits were sleep restricted compared with civilian life (52%; sleep restriction: 2.1 ± 1.6 h; Table 2) and 62% reported good PSQ. Recruits with sleep restriction (*vs* no sleep restriction) slept longer during civilian life, owing to later morning awakening, and slept less at the start of training (both *p* < .001; Table 2). Despite the sleep debt, at the start of training over half of recruits with sleep restriction reported good PSQ (58% *vs* 67% for no sleep restriction). Compared with the start, recruits experienced less sleep restriction during training (1.4 ± 1.3 h, *p* < .001; Table 2); nevertheless, sleep restriction classification tended to be consistent as 93% of recruits with no sleep restriction at the start of training experienced no sleep restriction during training and 71% with sleep restriction at the start endured sleep restriction. The proportion of recruits with a consistent PSQ classification at both the start and end of training was 71% for good and 43% for poor. A total of 93 physician diagnosed URTI episodes were recorded during training, of which 47% occurred during the first four weeks. Each URTI episode resulted in 3.3 ± 3.7 lost training days.

### Sleep restriction is independently associated with URTI

Unadjusted regression analysis showed that sleep restriction was independently associated with increased URTI susceptibility during the first four weeks of training (Figure 2A) and across the full 12-Weeks (model 1: OR = 2.99, 95% CI 1.35–6.65, *p* = .007). This association remained in the fully adjusted model accounting for sex, BMI, smoking, season, mood disturbance and long and short civilian sleep duration (model 8: OR = 2.93, 1.29–6.69, *p* = .011). Associations between sleep restriction and URTI across the full 12-Week course also remained in both unadjusted (Figure 2B) and adjusted models in recruits who completed training, accounting for a selection bias due to loss to follow-up (model 8: OR = 3.41, 1.09–10.67, *p* = .032). Moreover, those with sleep restriction at both the start and during training were more likely to suffer URTI (model 1: OR = 1.80, 95% CI 0.97–3.35, albeit *p* = .062). Poor PSQ at the start of training was not significantly associated with URTI; however, recruits with poor PSQ at both the start and end of training were more likely to suffer URTI across the full 12-Week course (model 8: OR = 3.16, 95% CI 1.31–7.61, *p* = .010).

### Good PSQ protects against the raised risk of URTI during sleep restriction

Hierarchical logistic regression showed that PSQ significantly moderated the association between sleep restriction and URTI susceptibility across the full 12 weeks (model 1: *B* = −1.12, SE 0.50, *p* = .023), whereby the association between sleep restriction and a raised risk of URTI was only observed in those with poor PSQ but not good PSQ. This moderating influence remained in the fully adjusted model accounting for sex, BMI, smoking, season, mood disturbance and long and short civilian sleep (model 8: *B* = −1.13, SE 0.50, *p* = .025). A moderating effect of PSQ on the association between sleep restriction and URTI susceptibility was also observed in the population who completed the training course, accounting for loss to follow-up (model 1: *B* = −1.28, SE 0.69, albeit *p* = .061).

Further analysis examined the combined influence of sleep restriction and PSQ on URTI susceptibility; with recruits who did not experience sleep restriction and reported good PSQ as the reference group (Figure 3A–B and Supplementary Table S3). Fully adjusted regression showed that recruits with no sleep restriction who reported poor PSQ at the start of training were at no greater risk of URTI in either the first four weeks (Figure 3A) or the full 12-Week course in those who completed training (model 8: OR = 0.65, 95% CI 0.20–2.04, *p* = .455; Supplementary Table S3). In contrast, recruits with sleep restriction who reported poor PSQ at the start of training were twice as likely to suffer URTI during the first four weeks of training (Figure 3A) and across the full 12-Week course in those who completed training (model 1: OR = 2.12, 95% CI 1.03–4.34, *p* = .040; Figure 3B and Supplementary Table S3). Despite a similar magnitude of sleep restriction in recruits reporting poor and good PSQ (poor 2.4 ± 1.3 h and good 2.7 ± 1.3 h during training; *p* = .152; Table 2), recruits with sleep restriction who reported good PSQ were at no greater risk of URTI in either the first four weeks or across the full 12-Week course in those who completed training (model 8: OR = 1.00, 95% CI 0.45–2.20, *p* = .999; Figure 3B and Supplementary Table S3). In summary, the independent association between sleep restriction and URTI was driven by recruits reporting poor PSQ; recruits with sleep restriction reporting good PSQ were at no greater risk of URTI compared with the reference group.

## Discussion

Meeting a one-size-fits-all recommendation for sleep duration (e.g. 7–9 hours’ sleep each night [10]) is unlikely necessary for all adults: individual sleep needs are likely an important consideration for optimal health [11, 12]. Empirical research shows that adults who habitually fall short of the recommended 7–9 hours’ sleep each night are more susceptible to respiratory infections [4–6], but the influence of restricting habitual sleep, now commonplace in modern society (e.g. rising early for work), and PSQ on respiratory infection incidence remains unknown. To this end, here we prospectively examined the association between sleep restriction, an individualized reduction in sleep duration ≥2 hours [31, 39], PSQ and physician diagnosed URTI in young adult male and female civilians embarking on a 12-Week military training course. Two new and noteworthy findings align with our hypotheses: first, sleep restriction was associated with increased URTI susceptibility during training (Figure 2A–B); second, and most notably, the observed association between sleep restriction and URTI was driven by recruits reporting poor PSQ. Fully adjusted regression analyses showed that, compared with recruits with no sleep restriction reporting good PSQ, recruits with sleep restriction at the start of training who reported poor PSQ were twice as likely to suffer URTI during the first four weeks of training, a time of heightened pathogen exposure, and during the full 12-Week course (Figure 3A–B and Supplementary Table S3). The likelihood of URTI was not significantly increased in sleep restricted recruits who reported good PSQ, despite a similar magnitude of sleep restriction during training in recruits reporting good and poor PSQ. Notwithstanding the inevitable impact of loss to follow-up reducing the number of respiratory infections available for comparisons, recruits enduring sleep restriction during training were more likely to suffer URTI. Moreover, recruits reporting poor PSQ when healthy at both the start and end of training were more than three times as likely to suffer URTI than recruits consistently reporting good PSQ. These findings show that sleep restriction is associated with increased URTI susceptibility, but only in recruits reporting poor not good PSQ.

The present findings are restricted to the level of association, and thus require cautious interpretation. Nonetheless, confidence in their significance is increased by rigorous steps taken including: excluding from analysis recruits with URTI symptoms at the time of or in close proximity to sleep assessments (i.e. mitigating reverse causation); showing consistent effects in recruits who not only completed training (i.e. accounting for selection bias due to loss to follow up) but also in recruits who endured sleep restriction during training (*vs* no sleep restriction) and in recruits who reported poor (*vs* good) PSQ at both the start and end of training. Regression models were adjusted for URTI risk factors including sex [43], BMI [44], smoking [45], and season [46], and further adjustments were made to account for long and short civilian sleep and mood disturbance, not only because these are likely URTI risk factors [5, 6, 36, 48] but also because of their known association with poor PSQ [11, 49–51]; for example, concordant are the present findings that recruits reporting poor PSQ also reported greater mood disturbance (Table 1). Confidence is increased that the observed associations with URTI susceptibility are not driven by mood disturbance because the data show comparable mood disturbance in recruits reporting poor PSQ with and without sleep restriction, yet URTI susceptibility was significantly greater than the reference group only in recruits with sleep restriction reporting poor PSQ (Figure 3A–B and Supplementary Table S3).

Candidate mechanisms to explain the observed associations between sleep restriction, PSQ and URTI require elucidation but likely include neuro-endocrine immune modulation [13] associated with circadian misalignment [52], subjective stress [53] and/or alterations in sleep architecture (e.g. SWS) [12, 13, 21]. In the present study, sleep restriction was largely due to consistently earlier morning awakening during military training than civilian life (Table 2), likely resulting in circadian misalignment, particularly for sleep restricted recruits. Our findings tentatively point to another explanation rather than circadian misalignment because bed and wake times during civilian life and military training were comparable in recruits with sleep restriction reporting good and poor PSQ (Table 2), yet URTI susceptibility was significantly greater (*vs* the reference group) only in sleep restricted recruits reporting poor PSQ. It is conceivable that sleep restricted recruits reporting poor PSQ experienced increased subjective stress and associated immune perturbations via disrupted HPA axis regulation of diurnal cortisol. Indeed, underground railway staff experienced a greater cortisol awakening response when rising early for a morning shift but only when the early shift coincided with poor PSQ and increased subjective stress [53]. Further research is warranted to explore these possibilities by adopting recommended measurements of circadian rhythm (e.g. melatonin [54]), subjective stress (e.g. perceived stress scale [55]), and HPA activity (e.g. diurnal cortisol [27, 56]).

We acknowledge that the present study has several limitations. The sample population comprised of young male and female infantry recruits, primarily Caucasian and of lower socioeconomic status (SES) [57], limiting the generalizability of these findings. Whether these findings are generalizable to other populations where sleep restriction is commonplace such as workers rising early for long commutes [58] and new parents [59] warrants investigation, particularly as low SES has been associated with increased vulnerability to URTI amongst short sleepers [60]. Nevertheless, civilians embarking on a military training course affords a unique opportunity to examine the association between sleep restriction, PSQ and URTI in a healthy, pre-screened population under standardized conditions. For example, although we did not assess physical training load and diet, both of which are considered to influence immune health [61, 62], these factors are relatively well controlled during military training because recruits perform largely the same daily physical activities and eat their meals from a limited menu at a military catering facility. We acknowledge that sleep was assessed in this study by self-report, e.g. PSQ was assessed using a single self-report item assessing the previous night’s sleep quality, and that objective sleep measures using actigraphy or polysomnography would have provided a more comprehensive sleep characterization. Having said this, self-reported sleep measures capture behaviors or perceptions that individuals can self-monitor, are practical for use in large population studies and relate to objectively assessed sleep [20, 21] and health outcomes [22, 23, 39]. The present finding that sleep restriction (defined as an individualized ≥ 2 h reduction in sleep duration) is associated with increased URTI susceptibility extends beyond those of previous studies, using the same sleep restriction threshold, showing that sleep restriction increased inflammation and cardiovascular disease risk [31, 39]; however, given the limited empirical data, research should further investigate the suitability of this sleep restriction threshold for neuro-endocrine immune and other health outcomes. As sleep was assessed only at the start and end of the training course, future research would also benefit from more regular sleep assessments to better characterize variations in sleep restriction and PSQ classifications across the measurement period. For example, although sleep restriction classification tended to be consistent at both time points in the present study, PSQ improved in over half of recruits who reported poor PSQ at the start of training. Despite this, confidence in the observed association between PSQ and URTI is bolstered by analysis showing that recruits reporting poor PSQ at both the start and end of training were more likely to suffer URTI (*vs* consistently good PSQ). Finally, future studies should confirm the infectious origin of physician diagnosed URTI by performing pathological analysis on throat swabs.

In conclusion, sleep restriction was associated with increased respiratory infection susceptibility during military training. However, the observed association between sleep restriction and respiratory infection was driven by recruits reporting poor PSQ; good PSQ was associated with protection against respiratory infection during sleep restriction. These findings advance our knowledge of how sleep restriction influences immune health, highlighting the need for future studies to account for individual sleep habits and sleep quality. A critical remaining knowledge gap, ripe for further enquiry, is whether improvements in sleep quality arising from behavioral sleep interventions translate to reduced respiratory infection during sleep restriction.

## Supplementary Material

zsac222_suppl_Supplementary_Material
