# Supplementary material for: Good perceived sleep quality protects against the raised risk of respiratory infection during sleep restriction in young adults
Source: Sleep. 2022 Sep 16;46(1):zsac222. doi: 10.1093/sleep/zsac222 (PMC9832516; doi:10.1093/sleep/zsac222)
Supplement: zsac222_suppl_Supplementary_Material [file zsac222_suppl_supplementary_material.docx]

**Good perceived sleep quality** **protects against the raised risk of respiratory infection during sleep restriction in young adults**

Neil P. Walsh^1^, Daniel S. Kashi^1^, Jason P. Edwards^1^, Claudia Richmond^1^, Samuel J. Oliver^2^, Ross Roberts^2^, Rachel M. Izard^3^, Sarah Jackson^4^ and Julie P. Greeves^4,5^

^1^Faculty of Science, Liverpool John Moores University, Liverpool, UK. ^2^College of Human Sciences, Bangor University, Bangor, UK. ^3^Defence Science and Technology, Porton Down, UK. ^4^Army Health and Performance Research, Army HQ, Andover, UK. ^5^Norwich Medical School, University of East Anglia, Norwich, UK.

**Corresponding author:**

Neil P. Walsh, Faculty of Science, Liverpool John Moores University, Liverpool, UK. Email: n.walsh@ljmu.ac.uk

| Supplementary Table 1. Descriptive information in complete cases and in the total sample following multiple imputation. | | | | | | |
| --- | --- | --- | --- | --- | --- | --- |
|  |  |  | | **Complete Cases** |  | **Total**  **Sample** |
|  |  | |  | ***N* = 1,107** |  | ***N* = 1,318** |
| *Demographic and lifestyle* |  | |  |  |  |  |
| Age |  | |  | 22 ± 3 |  | 22 ± 3 |
| Sex, Male [*N* (%)] |  | |  | 760 (69) |  | 898 (68) |
| Sex, Female [*N* (%)] |  | |  | 347 (31) |  | 420 (32) |
| Ethnicity, Caucasian [*N* (%)] |  | |  | 1051 (95) |  | 1253 (95) |
| BMI (kg/m^2^) |  | |  | 23.9 ± 2.7 |  | 23.9 ± 2.7 |
| Smoker [*N* (%)] |  | |  | 642 (58) |  | 777 (59) |
|  |  | |  |  |  |  |
| *Season of enrolment* |  | |  |  |  |  |
| Winter [*N* (%)] |  | |  | 129 (12) |  | 280 (21) |
| Spring [*N* (%)] |  | |  | 191 (17) |  | 214 (16) |
| Summer [*N* (%)] |  | |  | 392 (35) |  | 416 (32) |
| Fall [*N* (%)] |  | |  | 395 (36) |  | 408 (31) |
|  |  | |  |  |  |  |
| *Total mood disturbance* |  | |  | 4 ± 10 |  | 5 ± 9 |
|  |  | |  |  |  |  |
| *Sleep measures* |  | |  |  |  |  |
| Sleep duration during civilian life (h) |  | |  | 8.5 ± 1.6 |  | 8.5 ± 1.5 |
| Sleep duration at the start of training (h) |  | |  | 6.4 ± 0.8 |  | 6.4 ± 0.8 |
| Sleep restriction at the start of training (h) |  | |  | 2.1 ± 1.7 |  | 2.1 ± 1.6 |
| Sleep quality, Good [*N* (%)] |  | |  | 718 (65) |  | 820 (62) |
| Sleep quality, Poor [*N* (%)] |  | |  | 389 (35) |  | 498 (38) |
| Values presented as mean ± SD unless otherwise stated. BMI = body mass index. Sleep restriction is defined as an individualized reduction in sleep duration from civilian life. | | | | | | |

| Supplementary Table 2. Descriptive information in the population that completed training. | | | | | | | | | |
| --- | --- | --- | --- | --- | --- | --- | --- | --- | --- |
|  | **Total**  **Sample** |  | **No Sleep Restriction** | | |  | **Sleep Restriction** | | |
|  |  |  | All | Good PSQ | Poor PSQ |  | All | Good PSQ | Poor PSQ |
| *Population that completed training* | ***N* = 733** |  | ***N* = 365** | ***N* = 248** | ***N* = 117** |  | ***N* = 368** | ***N* = 218** | ***N* = 150** |
| *Demographic and lifestyle* |  |  |  |  |  |  |  |  |  |
| Age | 22 ± 3 |  | 22 ± 3 | 22 ± 3 | 22 ± 3 |  | 21 ± 3^bb^ | 21 ± 3^aa^ | 22 ± 3 |
| Sex, Male [*N* (%)] | 504 (69) |  | 284 (78) | 201 (81) | 83 (71)^a^ |  | 220 (60)^bb^ | 152 (70)^aa^ | 68 (45)^aabbcc^ |
| Sex, Female [*N* (%)] | 229 (31) |  | 81 (22) | 47 (19) | 34 (29)^a^ |  | 148 (40)^bb^ | 66 (30)^aa^ | 82 (55)^aabbcc^ |
| Ethnicity, Caucasian [*N* (%)] | 699 (95) |  | 343 (94) | 233 (94) | 110 (94) |  | 356 (97) | 209 (96) | 147 (98) |
| BMI (kg/m^2^) | 24.0 ± 2.7 |  | 24.1 ± 2.8 | 24.1 ± 2.9 | 24.3 ± 2.7 |  | 23.8 ± 2.6 | 23.9 ± 2.6 | 23.7 ± 2.5 |
| Smoker [*N* (%)] | 433 (59) |  | 219 (60) | 159 (64) | 60 (51)^a^ |  | 214 (58) | 135 (62) | 79 (53)^a^ |
|  |  |  |  |  |  |  |  |  |  |
| *Season of enrolment* |  |  |  |  |  |  |  |  |  |
| Winter [*N* (%)] | 127 (17) |  | 72 (20) | 43 (17) | 29 (25) |  | 55 (15) | 35 (16) | 20 (13)^b^ |
| Spring [*N* (%)] | 137 (19) |  | 64 (18) | 39 (16) | 25 (21) |  | 73 (20) | 42 (19) | 31 (21) |
| Summer [*N* (%)] | 189 (26) |  | 100 (27) | 71 (29) | 29 (25) |  | 89 (24) | 53 (24) | 36 (24) |
| Fall [*N* (%)] | 280 (38) |  | 129 (35) | 95 (38) | 34 (29) |  | 151 (41) | 88 (41) | 63 (42)^b^ |
|  |  |  |  |  |  |  |  |  |  |
| *Total mood disturbance* | 4 ± 8 |  | 3 ± 8 | 2 ± 8 | 5 ± 8^aa^ |  | 5 ± 9^bb^ | 4 ± 9 | 7 ± 9^aacc^ |
| Values presented as mean ± SD unless otherwise stated. PSQ = perceived sleep quality; BMI = body mass index. Sleep restriction is defined as an individualized reduction in sleep duration of ≥ 2 hours from civilian life.  ^a^ = significantly different to no sleep restriction Good PSQ; ^b^ = significantly different to equivalent no sleep restriction group. ^c^ = significantly different to sleep restriction Good PSQ. Single letter denotes *P* < 0.05 (e.g., ^a^); double letter denotes *P* < 0.01 (e.g., ^aa^). | | | | | | | | | |

| **Supplementary Table 3** Association between sleep restriction, PSQ and URTI during training. | | | | | | |
| --- | --- | --- | --- | --- | --- | --- |
|  |  | **No Sleep Restriction** | |  | **Sleep Restriction** | |
|  |  | Good PSQ | Poor PSQ |  | Good PSQ | Poor PSQ |
| ***Population that commenced training*** |  | OR | OR |  | OR | OR |
| *First 4 weeks* |  |  |  |  |  |  |
| Model 1: unadjusted (sleep restriction and PSQ) |  | *Reference* | 0.73 (0.23–2.32) |  | 1.15 (0.50–2.64) | 2.32 (1.07–5.02)^a^ |
| Model 2: sex + BMI |  | *Reference* | 0.70 (0.22–2.23) |  | 1.06 (0.46–2.46) | 1.95 (0.86–4.39) |
| Model 3: model 2 + smoking |  | *Reference* | 0.73 (0.23–2.34) |  | 1.09 (0.47–2.52) | 2.05 (0.91–4.64) |
| Model 4: model 3 + season |  | *Reference* | 0.74 (0.23–2.39) |  | 1.09 (0.47–2.54) | 2.02 (0.89–4.57) |
| Model 5: model 4 + total mood disturbance |  | *Reference* | 0.74 (0.23–2.38) |  | 1.09 (0.47–2.53) | 1.99 (0.87–4.59) |
| Model 6: model 5 + long sleep |  | *Reference* | 0.74 (0.23–2.38) |  | 1.06 (0.44–2.57) | 1.97 (0.84–4.60) |
| Model 7: model 5 + short sleep |  | *Reference* | 0.77 (0.24–2.51) |  | 1.03 (0.44–2.40) | 1.89 (0.82–4.35) |
| Model 8: model 5 + long + short sleep duration |  | *Reference* | 0.74 (0.23–2.39) |  | 1.11 (0.47–2.63) | 2.02 (0.87–4.68) |
|  |  |  |  |  |  |  |
| *12 weeks* |  |  |  |  |  |  |
| Model 1: unadjusted (sleep restriction and PSQ) |  | *Reference* | 0.56 (0.25–1.25) |  | 0.97 (0.56–1.69) | 1.68 (0.98–2.86) |
| Model 2: sex + BMI |  | *Reference* | 0.55 (0.24–1.22) |  | 0.95 (0.55–1.67) | 1.59 (0.91–2.80) |
| Model 3: model 2 + smoking |  | *Reference* | 0.56 (0.25–1.26) |  | 0.97 (0.55–1.70) | 1.65 (0.94–2.90) |
| Model 4: model 3 + season |  | *Reference* | 0.56 (0.25–1.26) |  | 0.97 (0.55–1.70) | 1.66 (0.94–2.93) |
| Model 5: model 4 + total mood disturbance |  | *Reference* | 0.57 (0.25–1.29) |  | 0.98 (0.56–1.73) | 1.71 (0.96–3.06) |
| Model 6: model 5 + long sleep |  | *Reference* | 0.57 (0.25–1.30) |  | 0.91 (0.50–1.65) | 1.63 (0.90–2.95) |
| Model 7: model 5 + short sleep |  | *Reference* | 0.57 (0.25–1.30) |  | 0.98 (0.55–1.73) | 1.71 (0.95–3.07) |
| Model 8: model 5 + long + short sleep duration |  | *Reference* | 0.57 (0.25–1.29) |  | 0.94 (0.53–1.67) | 1.67 (0.93–3.00) |
|  |  |  |  |  |  |  |
| ***Population that completed training*** |  |  |  |  |  |  |
| *12 weeks* |  |  |  |  |  |  |
| Model 1: unadjusted (sleep restriction and PSQ) |  | *Reference* | 0.55 (0.18–1.70) |  | 1.07 (0.50–2.26) | 2.12 (1.03–4.34)^a^ |
| Model 2: sex + BMI |  | *Reference* | 0.54 (0.17–1.66) |  | 1.07 (0.50–2.28) | 2.11 (0.99–4.48) |
| Model 3: model 2 + smoking |  | *Reference* | 0.58 (0.19–1.80) |  | 1.07 (0.50–2.29) | 2.19 (1.02–4.67)^a^ |
| Model 4: model 3 + season |  | *Reference* | 0.64 (0.20–2.00) |  | 1.04 (0.48–2.23) | 2.19 (1.02–4.72)^a^ |
| Model 5: model 4 + total mood disturbance |  | *Reference* | 0.65 (0.21–2.05) |  | 1.05 (0.49–2.26) | 2.26 (1.03–4.96)^a^ |
| Model 6: model 5 + long sleep |  | *Reference* | 0.65 (0.21–2.06) |  | 0.97 (0.43–2.18) | 2.15 (0.96–4.79) |
| Model 7: model 5 + short sleep |  | *Reference* | 0.65 (0.21–2.07) |  | 1.04 (0.48–2.25) | 2.24 (1.02–4.95)^a^ |
| Model 8: model 5 + long + short sleep duration |  | *Reference* | 0.65 (0.20–2.04) |  | 1.00 (0.45–2.20) | 2.20 (0.99–4.86) |
| PSQ = perceived sleep quality; URTI = upper respiratory tract infection; BMI = body mass index. Sleep restriction is defined as an individualized reduction in sleep duration of ≥ 2 hours from civilian life. Long sleep was defined as >10 hours civilian sleep duration. Short sleep was defined as <6 hours civilian sleep duration. **^a^** = *P* < 0.05. | | | | | | |
